# TEACHING MEDICAL STUDENTS TO INCORPORATE THE 4MS OF AGE-FRIENDLY HEALTH SYSTEMS ACROSS THE CONTINUUM OF CARE

**DOI:** 10.1093/geroni/igac059.130

**Published:** 2022-12-20

**Authors:** Jennifer Severance, Sarah Ross

**Affiliations:** UNTHSC-TCOM, Fort Worth, Texas, United States; University of North Texas Health Science Center, Fort Worth, Texas, United States

## Abstract

High quality and equitable health care for older adults involves patient-centered approaches that can improve the patient’s engagement, decision-making, and health outcomes. To educate future health professionals about patient-centered care across the continuum of care, a HRSA-Geriatric Workforce Enhancement Program created a four-week online elective course for third and fourth-year medical students using the Age-Friendly Health Systems 4Ms framework. Faculty identified geriatric tools and best practices in the 4M’s areas of What Matters, Mobility, Mentation, and Medication to develop self-directed asynchronous learning modules. Students were instructed on incorporating the 4Ms into patient assessments, care planning, interprofessional practice, and process improvement in different settings of care. Assignments included quizzes, case studies, discussion forums, and a patient experience interview with an older adult or caregiver. At course completion, students self-assessed their knowledge and skills and rated the course materials and assignments using five-point Likert-type items. Ninety-six students participated in the course between July 2020 and March 2022. Ninety-eight percent of respondents (n=80) felt the assignments were helpful, and 100% agreed the course improved their knowledge about Age-Friendly Health Systems and different care settings and processes. Also, 100% felt the content was applicable to their future practice and 96% would recommend the course. Qualitative thematic analysis of open-ended questions showed preferences for interactive elements, such as case presentations and guided interviews with older adults, as well as ways to improve student engagement. Educating students about Age-Friendly Health Systems can prepare future health professionals for collaborative patient-centered geriatrics care across a variety of settings.

